# The draining of capillary liquids from containers with interior corners aboard the ISS

**DOI:** 10.1038/s41526-021-00173-5

**Published:** 2021-11-11

**Authors:** Joshua McCraney, Mark Weislogel, Paul Steen

**Affiliations:** 1 Sibley School of Mechanical and Aerospace Engineering, Cornell University, Ithaca, NY 14850 United States; 2 Department of Mechanical and Materials Engineering, Portland State University, Portland, OR 97201 United States; 3 Smith School of Chemical and Biomolecular Engineering, Cornell University, Ithaca, NY 14850 United States

**Keywords:** Mechanical engineering, Aerospace engineering

## Abstract

In this work, we analyze liquid drains from containers in effective zero-g conditions aboard the International Space Station (ISS). The efficient draining of capillary fluids from conduits, containers, and media is critical in particular to high-value liquid samples such as minuscule biofluidics processing on earth and enormous cryogenic fuels management aboard spacecraft. The amount and rate of liquid drained can be of key concern. In the absence of strong gravitational effects, system geometry, and liquid wetting dominate capillary fluidic behavior. During the years 2010–2015, NASA conducted a series of handheld experiments aboard the ISS to observe “large” length scale capillary fluidic phenomena in a variety of irregular containers with interior corners. In this work, we focus on particular single exit port draining flows from such containers and digitize hours of archived NASA video records to quantify transient interface profiles and volumetric flow rates. These data are immediately useful for theoretical and numerical model benchmarks. We demonstrate this by making comparisons to lubrication models for slender flows in simplified geometries which show variable agreement with the data, in part validating certain geometry-dependent dynamical interface curvature boundary conditions while invalidating others. We further compare the data for the draining of complex vane networks and identify the limits of the current theory. All analyzed data is made available to the public as MATLAB files, as detailed within.

## Introduction

On earth, a hole in the bottom of a liquid-filled bucket is a convenient way to drain it. However, in the nearly weightless environment of orbiting or coast spacecraft, there is no “bottom” because there is effectively no gravity, and the liquid simply remains in the bucket. In fact, for many liquid handling operations aboard spacecraft, the phenomena are dominated by passive capillary forces over large length scales to which we are not accustomed. However, it is no less necessary to drain “buckets” aboard spacecraft; i.e., fuels, propellants, coolants, water. In terrestrial systems the effects of capillary forces have long been observed and exploited for sub-millimetric/micro-liter scale fluids processes where capillary forces are similarly dominant, including capillary origami^1^, bioprinting via elastic gels^2^, and lab-on-chip processes^3,4^. Especially in situations where the liquid involved is precious, it is important to process it in a manner that wastes nothing; i.e., one that achieves maximum drain rates with minimum liquid hold-up as a function of initial conditions, container geometry, and fluid properties.

In this paper we investigate the single port capillary draining of containers with interior corners, the interior corner serving as an open capillary conduit through which the liquid may be efficiently removed. Applications on earth and aboard spacecraft abound provided the flows are capillary-dominated as measured by the Bond number

1 $$\,{{\mbox{Bo}}}\,\equiv \frac{{{\Delta }}\rho g{H}^{2}}{\sigma } \,<\, 1,$$

where Δ*ρ* is the density difference across the interface, *g* the local acceleration field strength (i.e., gravity), *H* the characteristic length or height of the liquid along the interior corner, and *σ* the surface tension. Bo <1 is readily achieved aboard spacecraft where *g* ~10^−6^*g*_*o*_ is common. In low-gravity environments, the interior corner provides a measure of fluid stability^5^, say from residual accelerations, crew docking, and orbital maneuvers. In such cases *H* becomes the capillary length scale for the problem.

The Concus–Finn wetting condition^6^, which dictates the critical wetting conditions for a capillary flow to advance or recede depending on flow properties and corner geometry, specifies when a corner imbibes a liquid. While this work was mathematical, earlier heuristic critical wetting studies include bubble growth wetting^7^, meniscus stability in nucleation^8^, and drop condensation^9^. Through these works, tight arguments for idealized flow scenarios simplify the governing equations of motion to lubrication equations in corner flows^10,11^. Capillary corner flows have gained recent attention due to their prevalence aboard spacecraft for low-g liquid containment devices and have such been studied in myriad geometries including flows in propellant tank vane networks^12^, flow stability^13^, curved corners^14^, open rectangular channels^15^, and theoretical^16^ and experimental^17,18^ flows along nonplanar corners. Examples of short-duration low-g numerical investigations include single interior corners^19^, square channels^20^, stepped corners^21^, rounded corner walls^22^, and rounded vertices^23,24^. These investigations assume local parallel flow with negligible streamwise curvature and inertia as highlighted in earlier developments^25–27^. For example flows with non-negligible streamwise curvature and inertia we refer the reader elsewhere^28^.

For the typical liquids of terrestrial applications the critical length scale is the capillary length scale *H* ~(*σ*/*ρg*)^1/2^, which is sub-millimetric. However, in microgravity environments *H* can achieve sub-metric levels. The latter produces capillary flows and phenomena at unearthly large length scales with viscous-capillary time scales and inertial-capillary balances orders of magnitude above what is encountered on earth. As a consequence, due to the rarity of access to space, efforts are well-spent to extract as much quantitative data as possible from observed microgravity fluid behavior, a tack we pursue in this work.

In this paper we briefly review the story of NASA’s Capillary Flow Experiments conducted on the International Space Station over a 5-year period between 2010–2015. We sort through the many macro-capillary fluidic experiments performed, digitizing those experiments that provide fundamental flow data of relevance to the draining of precious liquids in containers with interior corners through single port locations. The work is plodding with each of the seven container types quantified in order. The data is useful for theoretical and numerical benchmarking, which we demonstrate using an established lubrication model. Good, adequate, and poor agreement in such comparisons provides directions for model acceptance or improvements, especially as regards the novelty of such low-g capillary flows with their centimetric viscous length scales and unusually high inertia.

## Methods

### Single drain experiments and data reduction

The capillary flow experiments (CFE) conducted aboard the International Space Station (ISS) by NASA were a series of handheld, centimetric-scale liquid experiments designed to probe capillary phenomena of fundamental and applied importance including contact line dynamics (CFE-CL, Contact Line), critical wetting in discontinuous structures (CFE-VG, Vane Gap), and passive ullage migration and bubble separations (CFE-ICF, Interior Corner Flow)^29,30^. Following completion of the experiments in flight, a NASA video archive for CFE was made publicly available at https://psi.nasa.gov/. The low-g flight data we analyse herein is mined from this resource.

In short, each CFE experiment required the partial filling of a certain container with liquid, observing the response of the liquid in low-g, and then simply draining that same liquid from the container. Approximately 2 to 3 h of crew time were required for each operation which included time to retrieve, set up, operate, and stow the hardware. Due to the rarity of observing such phenomena, even the filling and draining of such containers can be viewed as of both fundamental and applied interest. The CFE experiments were not designed with a quantitative study of capillary draining flows in mind. However, since such draining events were conducted as part of other procedures, their valued incidental nature is offset by their more qualitative character due to, say, manually-controlled drain rates as will be described.

**Fig. 1:**
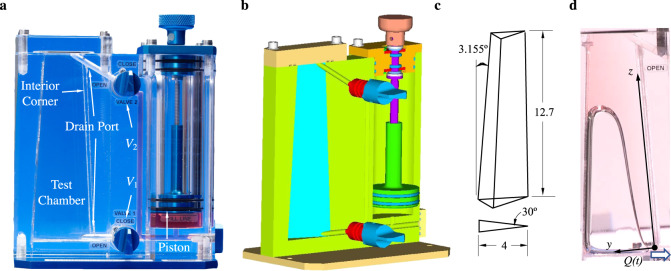
Annotated ICF-1 test vessel. Slender truncated 75-30-75 isosceles triangle-based pyramid with 3.155^∘^ taper. **a** Camera view of apparatus with valves *V*_1_ and *V*_2_ labeled, **b** solid model, **c** tapered test chamber wire model with dimensions in cm, where large-end (bottom) to small-end (top) cross-sectional isosceles triangles are congruent through a 20:13 ratio, and **d** cropped image of test chamber single drain operation aboard ISS. In addition to a superposition of the relevant coordinate system, **d** provides a bold blue arrow indicating drain direction at flow rate *Q*(*t*).

**Fig. 2:**
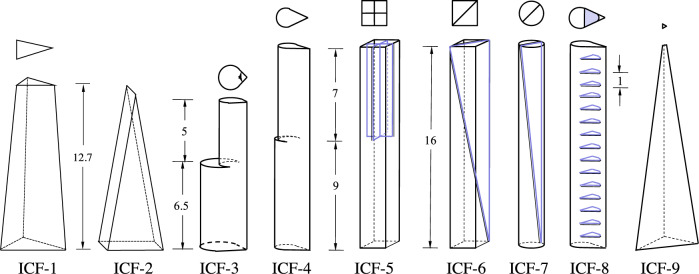
ICF test cells. Wire model sketches of ICF test cells with dimensions in cm.

We are interested in the capillary draining of containers with interior corners from a single exit port. Fig. 1 provides an image, solid model, and wire model for a typical drain test for CFE-ICF-1, for example. The breadth of the CFE-ICF experiment geometries is depicted using wire models in Fig. 2. Of the nine ICF experiments conducted (ICF-1, ICF-2, ..., ICF-9), seven single drain tests underwent single draining, and all are analysed and reported in detail herein. The results provide benchmarks for analytical and numerical methods predicting liquid drain rates and drain amounts from increasingly complex containers in low-g environments. All ICF tests employ perfectly wetting PDMS test fluids of select viscosity. (Note: Single drain tests imply the capillary draining of interior corners from one location, as opposed to doubly-drained interior corner tests which effectively employ two drain locations^31^. We also note that a single drain location often drains liquid through more than one interconnected interior corner).

**Fig. 3:**
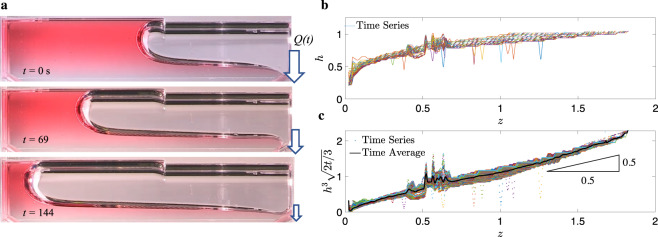
ICF single drain flow. **a** Time sequence of perfectly wetting *θ* = 0^∘^ red-dyed 2 cs PDMS in the ICF-4 test chamber drained through the “small-end” exit port at right. Note that the top image shows the ideal interface position at the drain port, the middle image shows gas ingestion, and the lower image shows an under-drained condition. **b**, **c** Dimensionless corner flow interface height profile *h*(*z*, *t*) for small-end drain tests for the ICF-4 test cell: **b** *h*(*z*, *t*) and **c** *h*^3^(2*t*/3)^1/2^ self-similar lubrication model solution with right triangle depicting slope of unity despite unequally scaled axes. The arbitrarily colored lines in **b** and dots in **c** are incremented at 1 Hz for the full 128 s run. In **b** the *h*(*z*, *t*) profiles decrease and extend with time, whereas in **c** they scatter about the time-averaged value (black line).

As our first test case, Fig. 1 provides an annotated image, solid model, and wire model sketch for the ICF-1 test vessel–a slender truncated 75-30-75 isosceles triangle-based pyramid. The test cell was originally designed for low distortion imaging of the flow along the 30^∘^ interior corner. For a partially filled vessel, the taper of the container provides a passive means of bubble migration and separation. A brief review of the drain procedures from a typical ICF vessel is as follows: The vessel was placed on an ISS work bench (MWA), back-lit by a diffuse light screen via cabin and portable lighting, and filmed via an HD Canon XF305 video camcorder. The test vessel was partially filled with liquid achieving a global low-g static equilibrium configuration. The astronaut then opened valve *V*_1_ or *V*_2_, often accessing the primary acute interior corner of the vessel, and drained the liquid into the reservoir by manually turning the piston dial counterclockwise. The dial was turned as quickly and continuously as possible trying not to ingest air at the drain port. Thus, a transient “maximum” drain rate from the test cell was established.

**Fig. 4:**
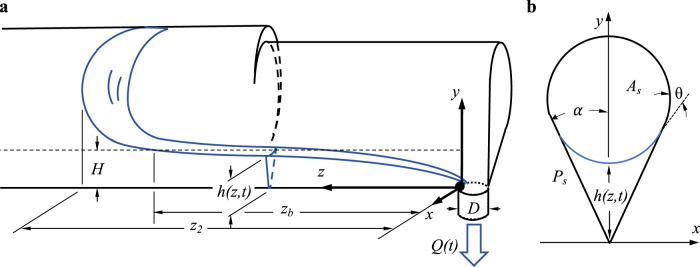
Single drain definition sketch. Single drain flow sketched for ICF-4: **a** Perspective profile and **b** cross-sectional view. Drain diameter *D* = 4 mm for all ICF test chambers.

Figure 3a provides images during the drain process for the ICF-4 test vessel. For this effectively constant cross-sectional area test cell drain scenario, as the liquid is removed, the bulk meniscus recedes, the interior corner flow length increases, and the overall maximum drain rate decreases. The capillary corner flow is driven by the capillary pressure gradient created at the exit port drain location. The astronaut works to drain the liquid as quickly as possible, establishing a nearly zero height meniscus at the drain location without ingesting gas. Transient fluid interface profile histories may be determined from such images. An annotated sketch of the flow modeled after ICF-4 is provided in Fig. 4 with profile and sectional views. The container geometry, fluid properties, and initial fill volume serve as the independent variables of the tests. The corner flow drain rates for ICF-1, -2, -3, -4, -6, -8, and -9 vessels are documented herein.

The ISS CFE-ICF drain test video data on the NASA archive is digitally reduced and analyzed herein, yielding time-dependent interface profiles, bulk meniscus receding velocities, and volumetric drain rates. As will be discussed in connection with the sketch of Fig. 4, the primary objectives are to digitize the fluid interface for the receding bulk meniscus *z*_2_(*t*) and interior corner height profiles *h*(*z*, *t*), while tracking time-dependent piston position to extract integral volumetric flow rates *Q*(*t*). For the data reduction, we employ in-house developed automated interface tracking algorithms to digitize the interface positions^32^. In brief, we first convert the video to low-loss still images and then reduce them to binary files. As indicated in Supplementary Fig. 1a for ICF-4, to reduce image noise due in part to small sparse air bubbles and spurious reflections, we apply a Canny filter^33^ applying two thresholds for edge detection sensitivity. We then increment the region of interest for each image and tabulate the desired interface pixels. This process is performed many hundreds of times for a total of 8442 seconds of low-g drain data over thousands of pixels for each still frame. The intermediate interfaces detected are then passed through a moving-average filter establishing the smoothed meniscus profiles *h*(*z*, *t*) traced, for example, *h*(*z*, *t*) in Supplementary Fig. 1b for ICF-3 (green line profile). As observed in Supplementary Fig. 1a, the presence of bubbles can pose challenges to the automated scheme, requiring either further filtering or manual tracking to determine *h*(*z*, *t*).

The HD video camcorder and ICF test vessel are manually positioned on the ISS work bench. Our digitized data are subsequently corrected for optical distortions arising from any misalignment and distortions caused by mismatched indices of refraction. The former could be shown to be truly negligible due to the ~1 m working distance of the camera, but ray trace analyses of the latter identified interface position corrections from 2% to nearly 10%. As a result, all ICF data sets are corrected for distortion due to diffraction.

### Reporting Summary

Further information on research design is available in the Nature Research Reporting Summary linked to this article.

## Results

### Lubrication theory overview

A current prediction of capillary draining from containers with interior corners exploits the lubrication approximation for slender flows^34^. We summarize the key details of the theory for comparisons to the newly reduced data. Figure 4a provides a sketch of the single drain problem for the ICF-4 container. The *z* axis runs along the interior corner vertex and defines the primary flow direction. For hydrophilic liquids satisfying the Concus–Finn corner wetting condition, as the liquid is removed at *z* = 0, a “filament” of liquid in the region *z* ∈ [0, *z*_*b*_] flows in the negative *z*-direction along the interior corner and out of the drain port as sketched in Fig. 4a and as shown in Supplementary Fig. 1a. The flow is primarily parallel, characterized by 1D cross-flow curvature as depicted in Fig. 4b. The liquid occupying *z* ∈ [*z*_*b*_, *∞*] is referred to as the bulk flow region with location *z*_2_ denoting the bulk meniscus advancing front. This region is characterized by quasi-steady 2D curvature. The dynamical boundary condition *H*(*z*_*b*_; *t*) marks the matching region between effectively 2D bulk and 1D corner flow regimes. During draining, along the corner the capillary pressure becomes increasingly negative as *z* approaches 0, since capillary pressure *P*(*z*) = −*σ*(1/*R*_1_ + 1/*R*_2_) ≈ −*σ*/*R* (low streamwise curvature implies *R*_2_ ≈ 0) where *R* = *R*(*z*, *t*) = *fh*(*z*, *t*) and *f* is a cross-flow interface curvature function. The resulting capillary pressure gradient drives liquid from higher to lower depths which are lowest at the drain port. Conserving volume, as the incompressible liquid is removed at the drain location the bulk meniscus advances (or recedes depending on your point of view) and the overlying gas expands as the pressure in the container decreases. Though neglected herein, we note that such decompression can lead to gas exchange across the liquid-free surface^35^.

In general^36^, we can assume a container geometry with *n* geometrically wetted interior corners satisfying the Concus–Finn condition. Let *j* serve as an index for a given wetted interior corner flow, where *j* = 1, 2, ... *n* for *n* total interior corners. Figure 4 depicts the purposely simplified ICF-4 container with a single *n* = 1 interior corner of half angle *α*_*j*_. While a variety of moving contact line models^37,38^ and subsequent solution techniques abound^39,40^, note here that the flow is largely parallel to the contact line allowing us to satisfactorily employ a constant contact angle *θ* condition. At long times *z*_2_(*t*) = *z*_*b*_(*t*) + *const* and from a priori predictions^41–43^ and a posteriori measurements^44^ it can be shown for quasi-static liquids in semi-infinite columns under microgravity conditions that *H*_*j*_(*z*, *t*) = *const* given by

2 $${H}_{j}=\frac{{P}_{s}\cos \theta }{2{{\Sigma }}{f}_{j}}\left(1-{\left(1-\frac{4{{\Sigma }}{A}_{s}}{{P}_{s}^{2}{\cos }^{2}\theta }\right)}^{1/2}\right),$$

where *P*_*s*_ is the perimeter of the container section, *A*_*s*_ is the container cross-sectional area, and Σ, *F*_*Aj*_, and *f*_*j*_ are the dimensionless normalized total wetted corner cross-sectional flow area, *j*th corner cross-sectional flow area, and *j*th corner cross-flow interface curvature functions, respectively:

3 $${{\Sigma }}=\mathop{\sum }\limits_{j=1}^{n}\frac{{F}_{Aj}}{{f}_{j}^{2}},\ \ \ {F}_{Aj}={f}_{j}^{2}\left(\frac{\cos \theta \sin {\delta }_{j}}{\sin {\alpha }_{j}}-{\delta }_{j}\right),\ \ \ {f}_{j}=\frac{\sin {\alpha }_{j}}{\cos \theta -\sin {\alpha }_{j}},$$

where *δ*_*j*_ ≡ *π*/2 − *α*_*j*_ − *θ*. *H*_*j*_ = *h*_*j*_(*z*_2_(*t*)) serves as the key dynamical boundary condition for the corner flow analysis. Because *H*_*j*_ is known as a function solely of the geometry and wetting conditions of the container, it is known a priori and used to scale the governing momentum equations. Thus, by choosing scales

4 $$z \sim L,\ \ \ h \sim H,\ \ \ t \sim {A}_{s}L/{Q}_{s},\ \ \ {Q}_{s} \sim \mathop{\sum }\limits_{j=1}^{n}{{F}_{A}}_{j}{{F}_{i}}_{j}{H}_{j}^{3}/L,$$

assuming a slender interior corner flow satisfying ${H}_{j}^{2}/{L}^{2}\ll 1$, the governing system reduces to a single dimensionless *z*-component visco-capillary momentum equation which may be solved along with the continuity equation to find solutions for what are henceforth denoted by dimensionless height *h*_*j*_(*z*, *t*) = *h*_1_(*z*, *t*) ≡ *h*(*z*, *t*), drain rate *Q*(*t*), and bulk meniscus advance rate *z*_*b*_(*t*). (We note that *F*_*i*_ = *F*_*i*_(*α*, *θ*) is a weak geometric viscous flow resistance function such that 1/8 ≤ *F*_*i*_ ≤ 1/6.) The *O*(1) self-similar solutions for such a single interior corner container are

5 $$h(z,t)={\left(z{(3/2t)}^{1/2}\right)}^{1/3},$$

6 $$Q(t)={(1/6t)}^{1/2},$$

7 $${z}_{b}(t)={(2t/3)}^{1/2}.$$

For weakly tapering (subscript *T*) nonconstant cross-section containers, the bulk meniscus height Eq. (2) may be approximated employing *A*_*s*_(*z*) and *P*_*s*_(*z*), yielding a *z*-dependent corner flow height condition where *h*_*j*_(*z*_*b*_(*t*), *t*) = *H*_*j*_(*z*_*b*_). For a linear taper as in the case of ICF-9, the *O*(1) analytic solutions for dimensionless height *h*_*T*_(*z*, *t*), drain rate *Q*_*T*_(*t*), and bulk meniscus advance rate *z*_*bT*_(*t*) for such container types are

8 $${h}_{T}(z,t)=H({z}_{bT}){\left(z/{z}_{bT}\right)}^{1/3},$$

9 $${Q}_{T}(t)={t}^{2}/27,$$

10 $${z}_{bT}(t)=t/3.$$

We measure and report the dimensionless interfacial height profiles *h*(*z*, *t*), volumetric flow rate *Q*(*t*), and bulk advancing front *z*_2_(*t*) as functions of time with each plot overlaid with the analytic solutions of Eqs. (5)–(7), or Eqs. (8)–(10), respectively. Test vessels ICF-1, -2, -3, -4, -6, -8, and -9 are reported below in numerical order for all single drain tests conducted on-orbit. The ICF-1, -2, -3, -4, and -6 vessels are longitudinally asymmetric. As such, draining from each end changes the characteristic geometric scales of the flow, effectively establishing two distinct experimental conditions for each test cell depending on which end is drained. Thus, there are two draining data sets for each test cell for vessels ICF-1, -2, - 3, -4, and -6.

Most of the ICF test cells were drained multiple times during the ISS experiments. We report all drains and denote the *i*th drain as ‘Run *i*’. Despite 60 fps video, all data were collected at either 1 or 0.2 Hz frequencies depending on the drain rate of the particular test. For any given run, hundreds of interface height profiles *h*(*z*, *t*) are digitized as shown in Fig. 3b for ICF-4. The analytical solutions are employed to collapse such data. For example, a rearrangement of Eq. (5) applied in Fig. 3c provides the favorable collapse indicated by the slope of unity (plot scale not 1-to-1). The favorable linear collapse of the data is clear despite noise due to the spurious digitization errors of the automated tracking algorithm. To filter the data into a single curve we time-average the collapsed data for each run generating a single interface profile curve as identified by the black line, say, in Fig. 3c. As time elapses, the bulk meniscus advances, increasing the *z*-domain over which *h*(*z*, *t*) is defined. This reduces the number of time-average samples at further downstream locations as time increases.

The experiments require a certain amount of time to establish something resembling a self-similar height profile. Only after this “start-up” period is it valid to compare the experiments with the long-time similarity solutions of the lubrication model. We thus introduce a time offset *t*_0_ for each flow. We calculate *t*_0_ as the time required for dimensionless *z*_*b*_ to advance from 0 to 1, or the time it would take for a fully-filled tank to drain until the measured bulk meniscus occupies the initial experiment volume recorded. Mathematically, this implies that *t*_0_ = 3/2 per Eq. (7) with *z*_*b*_ = 1 for constant cross-sectional vessels, and *t*_0_ = 3 per Eq. (10) for tapered conduits. Test cell ICF-8 is categorically unique and requires a different *t*_0_ value as will be discussed.

### ICF-1

An image, solid model, wire model, and big-end draining image were presented in Fig. 1 for the ICF-1 test vessel. This test cell was drained several times from each end (“small-end” and “big-end”). The section is a 75-30-75 isosceles triangle. The faces of this truncated triangular pyramid taper weakly at 3.155^∘^. Due to this shallow angle, the constant cross-sectional predictions of Eqs. (5)–(7) may be used to compare to the data collected for this geometry. The interface profiles *h*(*z*, *t*) are tracked at least to the intersection of *h*(*z*, *t*) with *H*(*z*) calculated from Eq. (2). The triangular section establishes *n* = 3 corner drains since all of the interior corners are critically wetted. Thus, three simultaneous interior corner flows take place during the single draining port process. The 75^∘^ corner flows bifurcate and flow along the even lower flow rate 90^∘^ corners formed by the base (big-end) or lid (small-end) before joining the draining flow at the exit port, which is aligned for imaging only the 30^∘^ corner flow in profile. Despite such complexities, the lubrication model for the three interior corner draining flows may be solved for comparisons to the data with expectations of over-prediction. The model is also employed to estimate that the two 75^∘^ corners together contribute ≈3% to the drain rate. Further details of the flow are listed in Table 1.

**Table 1:** ICF-1 fluid properties, scales, and constraints. Subscript 1 denotes the primary *α*_1_ = 30^∘^ corner.

Property	Units	ICF-1 small	ICF-1 big
Density, *ρ*	kg m^−3^	950	950
Viscosity, *μ*	kg m^−1^ s^−1^	0.019	0.019
Surface tension, *σ*	N m^−1^	0.0206	0.0206
Contact angle, *θ*	deg	0^∘^	0^∘^
Scales	Units	ICF-1 small	ICF-1 big
Half angle, *α*_1_	deg	15^∘^	15^∘^
Flow length, *L*	mm	49, 60, 46, 56	58, 58, 50, 36
Height, *H*_1_	mm	12.0, 11.5, 10.7, 11.1	11.6, 11.6, 11.9, 12.5
Perimeter, *P*_*s*_	mm	90, 87, 81, 84	88, 88, 90, 94
Surface area, *A*_*s*_	mm^2^	321, 299, 259, 277	302, 302, 318, 347
Geometry, ${{F}_{i}}_{1}$	-	0.141	0.141
Velocity, ${W}_{1}=\sigma {\sin }^{2}{\alpha }_{1}{{F}_{i}}_{1}/\mu {f}_{1}$	mm s^−1^	29.3	29.3
Flow rate, $Q=\mathop{\sum }\nolimits_{j = 1}^{3}{W}_{j}{{F}_{A}}_{j}{H}_{j}^{3}/L$	mm^3^ s^−1^	302, 222, 233, 213	234, 234, 293, 465
Time, *t* ~ *A*_*s*_*L*/*Q*	s	52, 81, 51, 73	75, 75, 54, 27
Time offset, *t*_0_	-	3/2	3/2
Lubrication assumptions	Constraint	ICF-1 small	ICF-1 big
Slender geometry, *ϵ*_1_ = *H*_1_/*L*	${\epsilon }_{1}^{2}\ll 1$	<0.05	<0.112
Capillary dominance	*Bo* ≪ 1	~10^−4^	~10^−4^
Low streamwise curvature	${\epsilon }_{1}^{2}{f}_{1}\ll 1$	<0.019	<0.04
Low intertia	${\epsilon }_{1}^{2}\rho \sigma {H}_{1}{\sin }^{4}{\alpha }_{1}/({f}_{1}{\mu }^{2})\ll 1$	<0.4	<1.0373
Low normal stress	${\epsilon }_{1}^{2}{\sin }^{2}{\alpha }_{1}\ll 1$	<0.0036	<0.008
Low saturation limit	*β*/(1 − *β*) ≪ 1	0.1516	0.1516
Static CL	${\epsilon }_{1}\beta {\sin }^{2}{\alpha }_{1}/{f}_{1}\ll 1$	<0.0059	<0.0087
Concus–Finn wetting	*θ* < 90^∘^ − *α*_1_	satisfied	satisfied

**Fig. 5:**
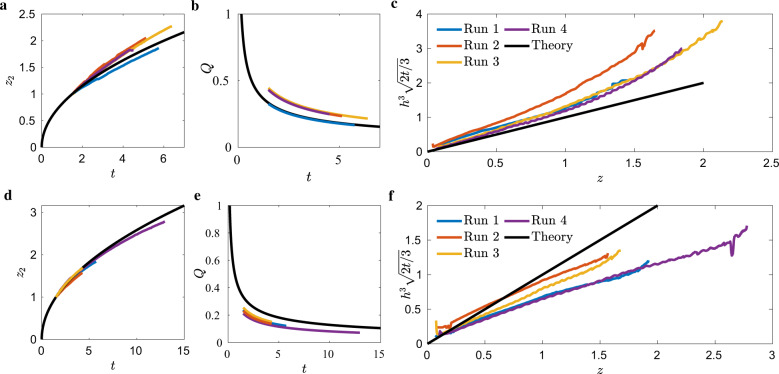
Reduced ICF-1 test cell drain data. Draining from small-end **a**–**c** and big-end **d**–**f**. **a** and **d** plot advancing bulk meniscus location *z*_2_(*t*), **b** and **e** volumetric flow rate *Q*(*t*), and **c**, **f** the self-similar time-averaged interface height profile ${h}^{3}\sqrt{2t/3}$, each overlaid with analytic solutions (black) of Eqs. (7), (6), and (5), respectively.

Figure 5a displays the ICF-1 results for the advancing bulk meniscus *z*_2_ for the case of small-end draining (*V*_1_ closed and *V*_2_ opened, Fig. 1a). Maximum discrepancies are 11% at the end of Run 2. The volumetric drain rates *Q* displayed in Fig. 5b are in qualitative agreement with the analysis. Quantitative comparisons are somewhat difficult to establish due to a certain lack of control of the drain rate. For example, gas ingestion at the drain exit port occurred in nearly all runs. When this occurred the astronauts would temporarily stop the draining and allow the passive capillary flow of liquid to displace the ingested gas, i.e., Fig. 3 (middle image). Furthermore, the large piston diameter of 38 mm leads to a fairly insensitive measure of flow rate based on time-dependent piston location and flow rate on the order of 220 μm/s. The latter point is addressed by fitting a curve to the piston location, such as Supplementary Fig. 2a and time-differentiating the smoothed curve, rather than differentiating the discrete piston locations, Supplementary Fig. 2b. Nonetheless, we report volumetric flow rate average uncertainties <28% for all runs. Draining ICF-1 from the small-end allows *H*(*z*) to increase with *z*. As such, the meniscus is expected to overshoot the constant height solution computed assuming a constant cross-section. Figure 5c confirms this, as all four test runs overshoot the constant height prediction. Despite the overshoot, data still scales with Eq. (5) and maintains the same order of magnitude.

Figure 5d presents *z*_2_(*t*) for draining from the big-end of the ICF-1 test cell (*V*_1_ opened and *V*_2_ closed, Fig. 1a). Maximum discrepancies are 7% by the end of Run 2. Similar to the small-end drain, *Q* follows the theory fairly well considering the aforementioned experimental complications, agreeing within <22%. Draining ICF-1 from the large-end allows *H*(*z*) to decrease with *z*. Consequently, *h*(*z*, *t*) is expected to undershoot the constant height solution as observed in Fig. 5f.

### ICF-2

**Fig. 6:**
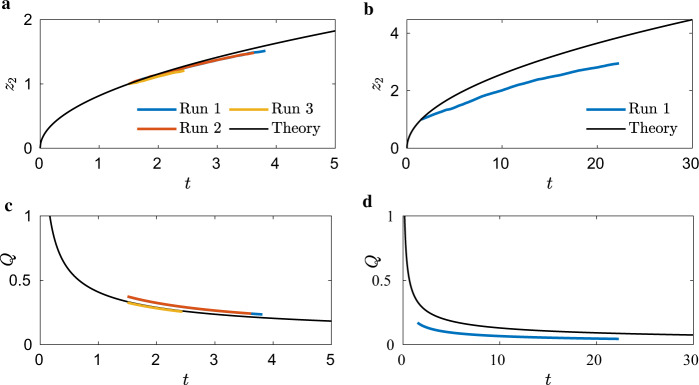
ICF-2 data. ICF-2 advancing bulk meniscus location *z*_2_(*t*) and volumetric flow rate *Q*(*t*). **a**, **c** small-end drain and **b**, **d** large-end drain.

The ICF-2 test cell was similarly drained from small- and big-ends. Though the ICF-2 test cell side walls taper at 8.95^∘^, it can be shown that the impact of this taper is weak on the flow and that the height condition quickly establishes a nearly constant value of *H*(*z*) ≈ *D*/2*f* = 0.207*D* (where *D* = 23 mm), which varies <10% for the flow. Thus, again the drain data collected for ICF-2 may be best compared to the constant cross-sectional predictions of Eqs. (6), (7). As highlighted in Supplementary Fig. 4, the rectangular cross-section establishes *n* = 4 simultaneous interior corner flows. The ICF-2 values are tabulated in a similar manner as in Table 1 for ICF-1, see Supplementary Table 1. Figure 6a presents *z*_2_ drained from the small-end (*V*_1_ closed and *V*_2_ opened, Supplementary Fig. 4a), which are nearly coincident, showing maximum error with the lubrication prediction within 5%. Figure 6c presents *Q*(*t*), the summation of the four corner flows, which agrees with the lubrication model within 12%. Corner flow height profiles *h*(*z*, *t*) for ICF-2 can not be digitized for this test cell due to the non-orthogonal viewing angle as observed from Supplementary Fig. 4d. The big-end (*V*_1_ opened and *V*_2_ closed, Supplementary Fig. 4a) was drained only once. The results of this test for *z*_2_ and *Q* are shown in Fig. 6b and d, where only qualitative agreement is observed with the model predictions.

### ICF-3

**Fig. 7:**
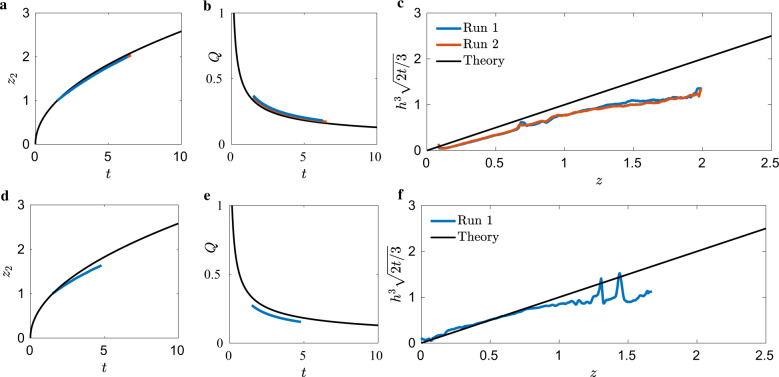
ICF-3 data. ICF-3 test cell drained from **a**–**c** small- and **d**–**f** big-ends. **a**, **d** advancing bulk meniscus location *z*_2_(*t*), **b**, **e** volumetric flow rate *Q*(*t*), and **c**, **f** the self-similar time-averaged interface height profile ${h}^{3}\sqrt{2t/3}$, each overlaid with theory (black), Eqs. (7), (6), and (5), respectively.

The ICF-3 test cell was also drained from both small- and big-ends. During draining, the advancing front was drained while only residing within a constant cross-section of the test cell–the step geometry was not encountered. The data were thus compared to the constant cross-sectional predictions of Eqs. (5)–(7). The snow-cone cross-section sketched in Supplementary Fig. 5 implies *n* = 1 edge. Additional data for these tests are provided in Supplementary Table 2. Figure 7a presents results for the advancing front *z*_2_ drained from the small-end (*V*_1_ closed and *V*_2_ opened, Supplementary Fig. 5a), and while only two runs were conducted, the advancing front of each is nearly coincident. Excellent agreement with the lubrication prediction is observed, of which the maximum error of both runs is <3.5%. Figure 7b presents *Q*(*t*), with maximum errors for both runs <12%, which is favorable agreement considering the limitations discussed in section ICF-1. The self-similar analytic interfacial height over-predicts the observed height. This could be explained via inertial effects, which are not necessarily small for this test.

Similar results are observed for the ICF-3 big-end drain (*V*_1_ opened and *V*_2_ closed, Supplementary Fig. 5a). Figure 7d presents *z*_2_ as a function of time, which is over-predicted by the lubrication prediction with maximal error <8.5%. This could be due to the volumetric flow rate, which is also over-predicted by the theory on average <14%, Fig. 7e. The self-similar analytic interfacial height shows excellent agreement for domains near the drain, but as the advancing front continues, downstream heights are increasingly over-predicted by the theory, Fig. 7f. Again, the large inertia of these test might explain this observation.

### ICF-4

**Fig. 8:**
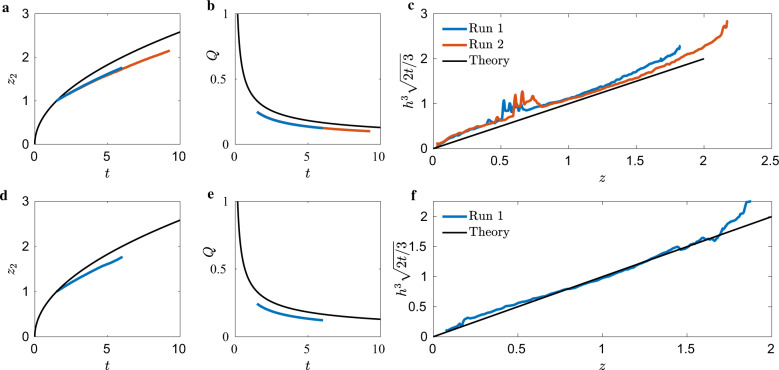
ICF-4 data. **a**–**c** Small-end and **d–f** big-end drain. **a**, **d** present advancing bulk meniscus location *z*_2_(*t*), **b**, **e** volumetric flow rate *Q*(*t*), and **c**, **f** the self-similar time-averaged interface height profile *h*^3^(2*t*/3)^1/2^.

The ICF-4 test cell was also drained from each end. A constant cross-section was maintained during draining–the step geometry was not encountered. The data is thus compared to the constant cross-sectional predictions of Eqs. (5)–(7). The ice-cream-cone cross-section of Supplementary Fig. 6 implies *j* = 1. Figure 8a presents *z*_2_ alongside the theoretical prediction, with maximum error <14% for the small-end draining (*V*_1_ closed and *V*_2_ opened, Supplementary Fig. 6a). Additional data for these tests are provided in Supplementary Table 3. The volumetric flow rate follows the theoretical curve for later times but is nearly constant for the duration of the runs. Figure 8c plots the self-similar interfacial height profiles. Noise is measured in regions *z* ∈ [0.5, 1], where unfavorable lighting complicates interface tracking. Regardless, the theory under-predicts *h*(*z*, *t*), which is not explained by the relatively high inertia. However, the volumetric flow rate is less than the theoretical flow rate, Fig. 8b. The theory assumes zero height at the drain, but in many runs, the astronauts were only able to reduce drain height to between 2 to 3 mm without ingesting gas (see drain port details, Fig. 4). With average drain rates <23% in the prediction, it is expected the meniscus heights are lower, and the advancing front is slower, both of which are observed.

Big-end drain (*V*_1_ opened and *V*_2_ closed, Supplementary Fig. 6a) results are similar and Fig. 8d presents *z*_2_ against Eq. (7) with errors <13%. The volumetric flow rate shown in Fig. 8e exhibits qualitative agreement to theory <27%, as discussed in section ICF-1. For this run, the lubrication prediction accurately predicts meniscus interfacial height, with an average error <9%, Fig. 8f.

### ICF-6

**Fig. 9:**
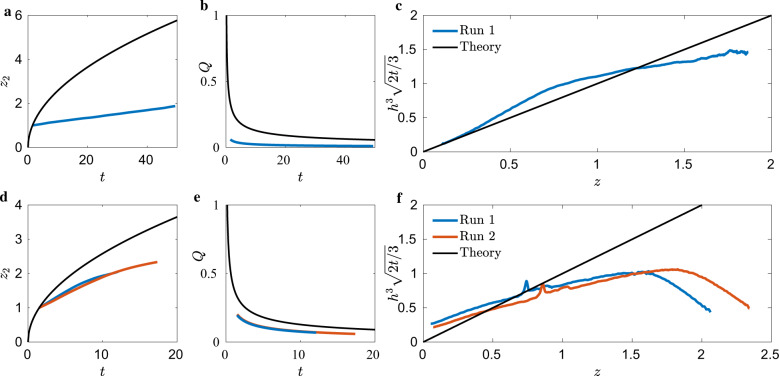
ICF-6 data. Draining from **a**–**c** small- and **d**–**f** big-ends. **a**, **d** present advancing bulk meniscus location *z*_2_(*t*), **b**, **e** volumetric flow rate *Q*(*t*), and **c**, **f** the self-similar time-averaged interface height profile *h*^3^(2*t*/3)^1/2^.

The ICF-6 test cell was also drained from each end. The linearly tapered diagonal vane runs the length of the test cell establishing significantly different drain geometries depending on the end drained, Supplementary Fig. 7a. The end with the zero vane height is referred to as the big-end and the end with the vane height that spans the diagonal is referred to as the small-end. In both drain scenarios, the bulk meniscus is assumed to advance through a constant cross-section, though it is clearly complicated by the presence of the pinning edges of the vane for varying portions of the flow. Such conditions are not addressed by the lubrication model. As sketched in Supplementary Fig. 7c, the square section contains four corners, but the vane divides the section into two 45-90-45 isosceles triangles with primary drain path along the *α*_1_ = 22.5^∘^ interior corner formed by the vane and side wall, where the 1 mm thick vane bisects each drain port. The drain detail in Supplementary Fig. 7c illustrates how the drain port spans the diagonal vane such that fluid is simultaneously removed from both sides of the vane. Though the theory is inappropriate for this test cell, we apply it anyway. We model the flow with *n* = 5 edges: two 45^∘^ corners (*α*_1_ = 22.5^∘^) and three 90^∘^ corners (*α*_2_ = 45^∘^), for a total flow rate of *Q* = 2*Q*_1_ + 3*Q*_2_. Additional data for these tests are provided in Supplementary Table 4. We note that 2*Q*_1_ is ~30 times larger than 3*Q*_2_, implying that the flow is dominated by the two 45^∘^ vane corners, Fig. 9d. Figure 9a shows the bulk meniscus as drained from the big-end (*V*_1_ opened and *V*_2_ closed, Supplementary Fig. 7a), which is over-predicted by poorly equipped Eq. (7). The bulk meniscus is nearly linearly in time, which suggests the vane acts as a tapered geometry though significantly slower than the 1/3 temporal slope, Eq. (10). The volumetric flow and bulk meniscus advance rates are significantly less than the theoretical predictions, Fig. 9b. This suggests that the impact of the contact line pinning along the vane edge is to flatten the interface corner menisci effectively increasing the contact angle in turn reducing the capillary pressure gradient and flow velocity. These flows are clearly complicated by contact line pinning along the vane edge as the vane height decreases, which is not currently accounted for in the theory. An effort to derive a more representative set of dynamical boundary conditions for *H*(*z*) may prove fruitful.

The ICF-6 small-end drain (*V*_1_ closed and *V*_2_ opened, Supplementary Fig. 7a) bulk meniscus *z*_2_ is also seen to undershoot the lubrication theory, Fig. 9d. Again, large effectively pinned contact angles in part explains the slower advancing front, Fig. 9e. But in this case, in particular, the meniscus height *h*(*z*, *t*) and bulk meniscus height *H*(*z*_*b*_, *t*) are effectively held down by vane edge pinning. A special application of Concus–Finn theory to establish tapered vane pinned contact line conditions and an improved *H*(*z*_*b*_, *t*) would be required to make the lubrication model more quantitatively applicable for this “weakly tapered” test cell type.

### ICF-8

**Fig. 10:**
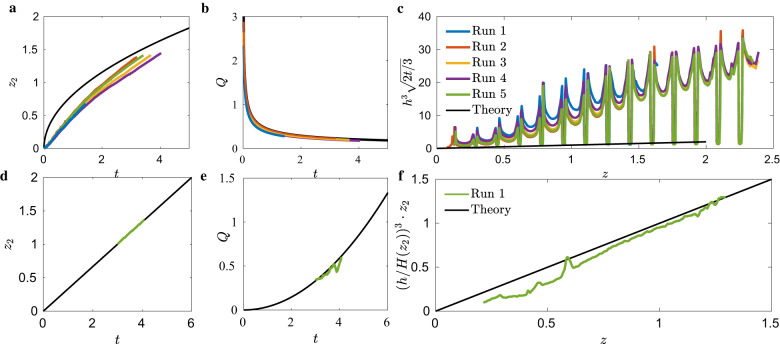
ICF-8 and -9 data. Single drain data for ICF-8 and -9 cells. **a**–**c**: ICF-8 drain data. **a** Bulk advancing front *z*_2_, **b** volumetric flow rate *Q* plotted against time, and **c** self-similar time-averaged interfacial height *h*^3^(2*t*/3)^1/2^, each overlaid with analytic solutions (black) from Eqs. (5), (6), and (7), respectively. **d**–**f**: ICF-9 drain data in the linearly tapered section. **d** Bulk advancing front location *z*_2_(*t*) and **e** volumetric flow rate *Q*(*t*), and **f** the self-similar time-averaged interfacial height with the respective analytic solutions of Eqs. (10), (9), and (8) (black).

Supplementary Fig. 8 provides an image, solid model, and wire model for the ICF-8 test vessel. The test cell is a constant ice-cream-cone section interrupted by vane partitions that divide the interior corner region incrementally in the primary flow direction while allowing flow through an opening near the vertex of the interior corner. We note that the height of the opening is approximately equal to the constant height boundary condition *H* for the ice-cream-cone section container without the partitions. In the absence of any other analytical model for this geometry, we again employ the constant cross-section lubrication approximations of Eqs. (5)–(7) in remote hopes of qualitative assessment. Unlike the other drain tests, the ICF-8 data does not account for an initial time offset *t*_0_ and the flow does not conform to the negligible streamwise curvature assumption of the lubrication theory. We apply it nonetheless. The ice-cream-cone cross-section (Supplementary Fig. 8c) implies *n* = 1. The bulk meniscus initially advances slower than the predictions, but it is clear given longer drain times the bulk meniscus would overtake the lubrication prediction due to non-negligible streamwise curvature and the liquid wetting the partitions. From Fig. 10c it is observed how poorly the liquid profile *h*(*z*, *t*) is captured by the analysis, the discrete non-negligible streamwise curvature maintaining levels far in excess of the Concus–Finn theory predictions for *H*(*z*_*b*_). Surprisingly, volumetric flow rates favorably agree with the lubrication prediction within 2%, as shown in Fig. 10b. We conjecture that this potentially coincidental finding may be due to the fact that streamwise curvature is nearly constant and equal for all vane segments and that the viscous resistance of the flow is governed by *H* of the vane gap and not the actual *H* of the flow. Additional data for these tests are provided in Supplementary Table 5.

### ICF-9

The fully tapered geometry of ICF-9 shown in Supplementary Fig. 10c establishes a flow geometry that unquestionably satisfies the tapered container drain theory, Eqs. (8)–(10). The equilateral triangular cross-section achieves *j* = 3. Figure 10d presents the advancing front *z*_2_(*t*) data, with draining from the small-end (*V*_1_ closed and *V*_2_ opened, Supplementary Fig. 10a). Unlike all other weakly tapered test cells, ICF-9 exhibits a bulk meniscus that advances linearly in time, which agrees with theoretical predictions within a maximum error of <1%, as shown in Fig. 10e. As suggested by the theory, the maximum volumetric flow rate increases in time, unlike all other runs, with maximum error <30% and average error <10%. The interfacial height also compares favorably to the analytic solution Eq. (8), with an average error <20%, Fig. 10f. The ICF-9 test cell satisfies the assumptions of the model to the greatest extent with exceptionally low inertia up to two orders of magnitude lower than the other test cells. Additional data for this test is provided in Supplementary Table 6.

## Discussion

Altogether, we find that many of the “incidental” drain tests performed by the astronaut crews for the CFE-ICF experiments on ISS provide rare data of value for theoretical and numerical benchmarks. By digitizing all single drain events, over 2 h of flight data reported, from the CFE-ICF space experiments we are able to establish a limited, though rare, data set for such draining flows in containers with interior corners. We find that the experimental results are predicted fairly well by a simple zero-gravity quasi-steady lubrication analysis.

In this 5-year post-flight data reduction investigation, a total of 27 experiment runs for seven of the nine CFE-ICF test vessels are mined and presented herein from the NASA PSI archive https://psi.nasa.gov/ for the draining flow phenomena in containers with interior corners. Referring to Fig. 2, several families of drain geometries are represented including tapering triangular sections (weak taper ICF-1 and strong taper ICF-9, both with *n* = 3 wetted corners), tapering rectangular sections (ICF-2 with *n* = 4 wetted corners), stepped tapers (ICF-3 and -4 with *n* = 1 wetted corners), square sections with tapered vanes (ICF-6 with *n* = 5 wetted corners), and partitioned constant cross-sections (ICF-8 with *n* = 1 wetted corners). As a demonstration of the value of the data, we employ it to benchmark a simple lubrication model. The model assumes slender capillarity-driven flows along interior corners with negligible streamwise curvature and dynamical interface height boundary conditions (i.e., *z*_2_(*t*), *H*(*z*), etc.). We assess the validity of the model assumptions applicable to each of the ICF draining flow geometries while comparing the predictions with the data for flow rate *Q*(*t*), advancing bulk meniscus *z*_2_(*t*), and corner flow profile *h*(*z*, *t*) when observable. Although the analysis might be tuned to each unique draining flow, our rather cavalier comparisons show average agreement with flow rates to within ±21% and advancing bulk menisci to within ±8% for the test cells conforming to theory, ICF-1, -2, -3, -4, -9. Specific discrepancies may be blamed on irregular manually induced draining mechanisms, finite meniscus height conditions at the drain location, non-negligible inertia, boundary condition violations, and the presence of spurious expanding bubbles. These deviations observed outline the direction of further theoretical work to address such geometric complexities as planar and curved wall interior corner, vane edge pinning boundary conditions, and local streamwise curvature, to name a few. Nonetheless, it appears the lubrication model provides an adequate prediction of interior corner flow draining in the low-g environment provided the flows are slender. Such predictions are valued for precious liquid recovery from containers and conduits in large-scale systems aboard spacecraft and small-scale systems on earth. Numerical benchmarks may be similarly pursued using the present data set.

The capillary drain data presented is extracted from the NASA archive for CFE, which was conducted on ISS over the 2010–2015 time frame. The archived, publicly-accessible image data is corrected for misalignment and diffraction and smoothed as necessary to provide a benchmarking data set for further theoretical and numerical model development, as well as for practical fluids system design. The effort demonstrates the untapped value of the image data stored on the NASA online archives.

## Supplementary information

Reporting Summary

Supplementary Information

## Data Availability

The discretized data may be found at figshare public data repository here: https://figshare.com/articles/dataset/Single_Drain_Data/16632472^45^. An explanation of the data is provided in the Supplementary Data Tables and Data Usability Description.

## Code availability

The image tracking algorithms employed to track interface and piston locations may be accessed by contacting the authors.
