# Publisher Correction: The draining of capillary liquids from containers with interior corners aboard the ISS

**DOI:** 10.1038/s41526-021-00181-5

**Published:** 2021-12-14

**Authors:** Joshua McCraney, Mark Weislogel, Paul Steen

**Affiliations:** 1 Sibley School of Mechanical and Aerospace Engineering, Cornell University, Ithaca, NY 14850 United States; 2 Department of Mechanical and Materials Engineering, Portland State University, Portland, OR 97201 United States; 3 Smith School of Chemical and Biomolecular Engineering, Cornell University, Ithaca, NY 14850 United States

**Keywords:** Mechanical engineering, Aerospace engineering, Mechanical engineering, Aerospace engineering

Correction to: *npj Microgravity* 10.1038/s41526-021-00173-5, published online 11 November 2021

In this article Figures 1, 3 and 4 were cited out of order. Figure 3 was published as Figure 1, Figure 4 was published as Figure 3, and Figure 1 was published as Figure 4. Captions were positioned correctly. The original article has been corrected.

