# Supplementary material for: The draining of capillary liquids from containers with interior corners aboard the ISS
Source: NPJ Microgravity. 2021 Nov 11;7:45. doi: 10.1038/s41526-021-00173-5 (PMC8585966; doi:10.1038/s41526-021-00173-5)
Supplement: Supplementary file 2 — Supplementary Information [file 41526_2021_173_MOESM2_ESM.pdf]

Supplementary Data Tables and Data Usability Description

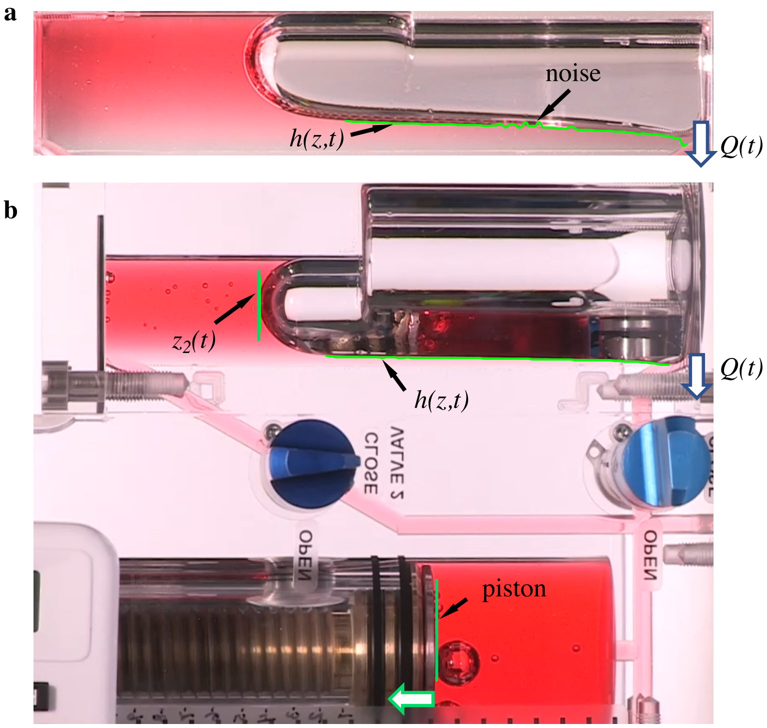

Supplementary Figure 1: Images of ICF single interior corner drain tests: **a** ICF-4 Test Cell only and **b** ICF-3 full test vessel. Blue arrows denote volumetric flow rate drain locations and green arrows denote piston position and direction of motion. Green lines identify meniscus profiles $h(z,t)$, bulk meniscus location $z_2(t)$, and piston location.

Supplementary Figures 5-9 provide a test cell image, solid model, and wire model, and photograph of single drain for the various vessels studied.

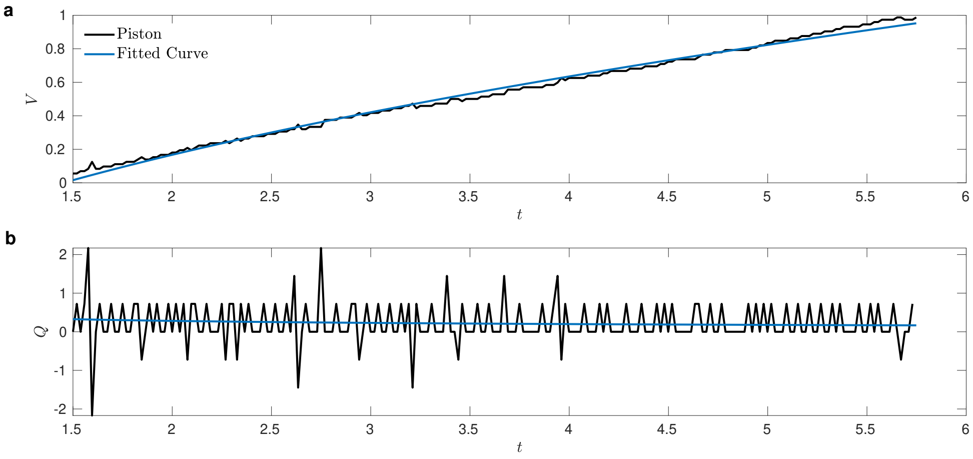

Supplementary Figure 2: **a** Drain volume V (black) (akin to piston location) and fitted curve (blue). **b** measured volumetric flow rate dV/dt (black) and temporal derivative of fitted curve (blue).

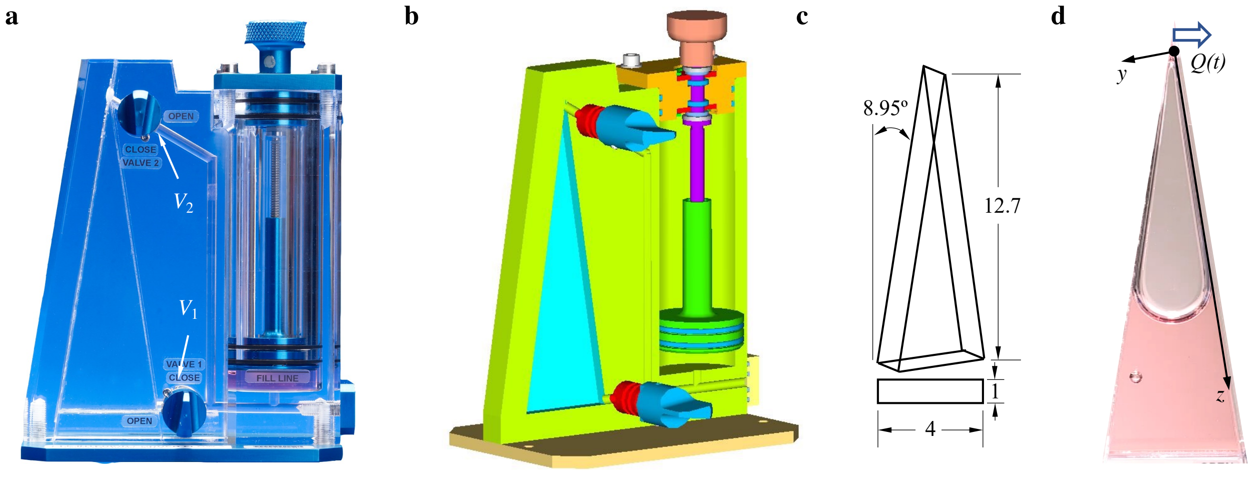

Supplementary Figure 3: ICF-2 test vessel: Tapered rectangular section. **a** Test vessel, **b** solid model of the test cell, **c** tapered test cell wire model with dimensions in cm, and **d** cropped image of small-end drain test. In addition to a superposition of the relevant coordinate system, **d** provides bold blue arrow indicating drain direction at flow rate Q.

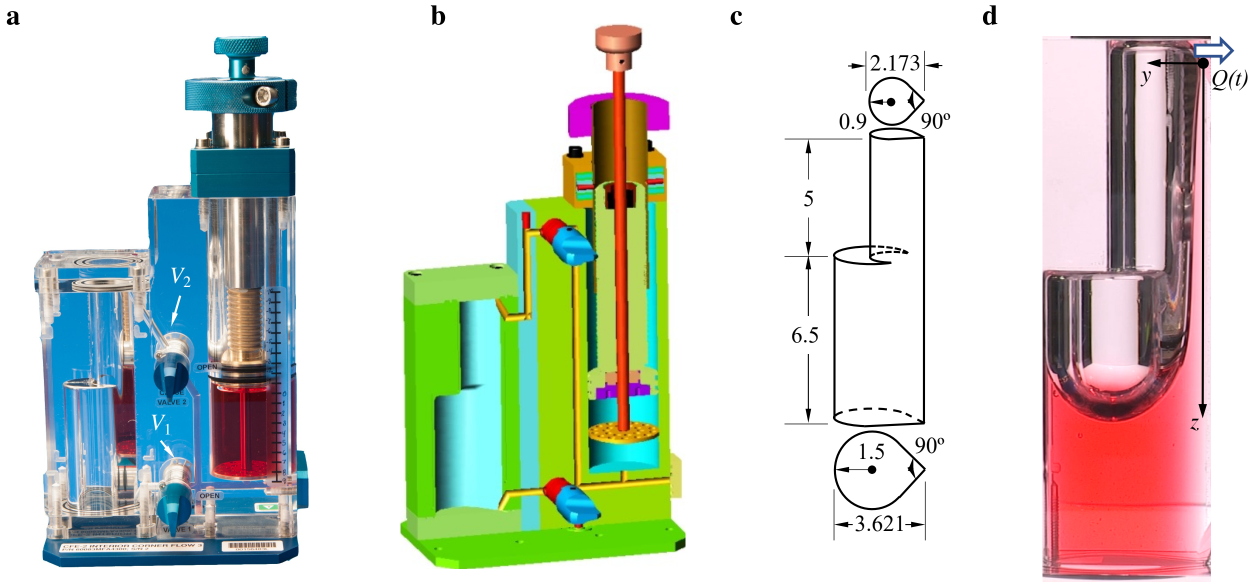

Supplementary Figure 4: ICF-3 test vessel: ice-cream-cone test cell with large stepped taper. **a** test vessel, **b** solid model, **c** test cell wire model with dimensions in cm, and **d** cropped image of test cell single drain operation.

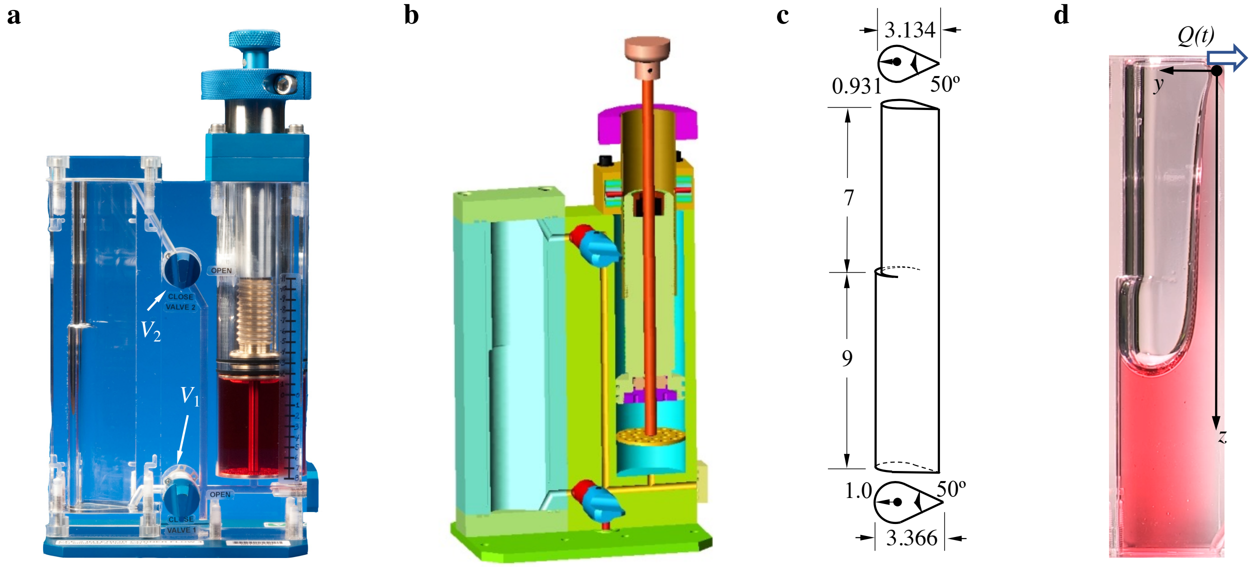

Supplementary Figure 5: ICF-4 test vessel: ice-cream-cone section with small stepped taper. **a** Test vessel, **b** solid model, **c** enlarged test cell wire model with dimensions in cm, and **d** cropped image of test cell single drain operation.

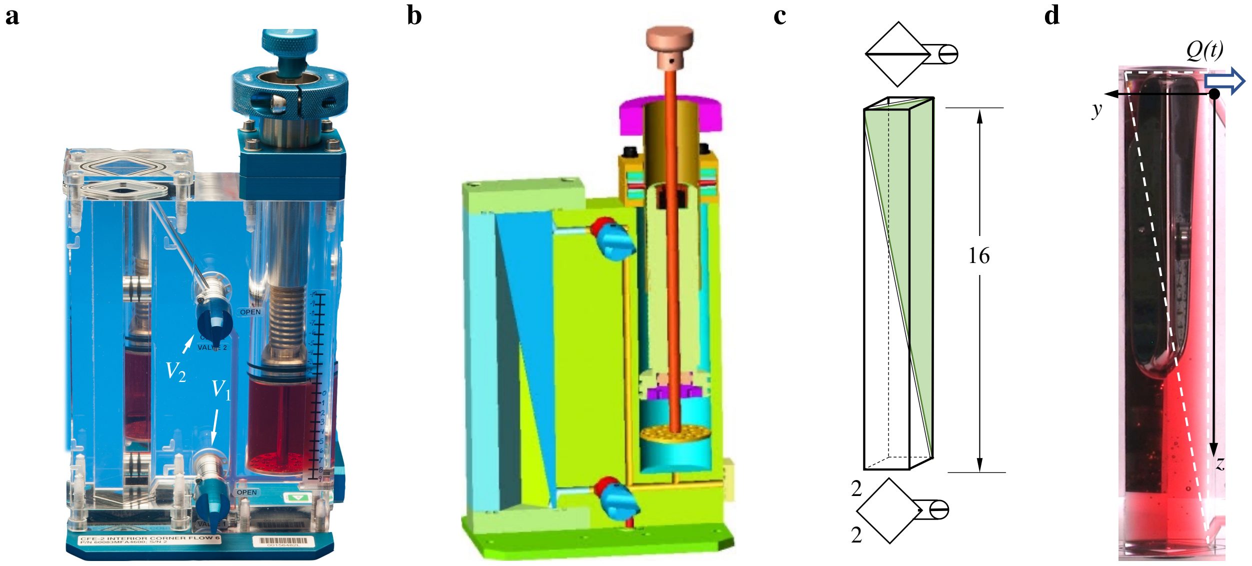

Supplementary Figure 6: ICF-6 test vessel: square section with diagonal linearly tapered vane. **a** Test vessel, **b** solid model, **c** enlarged test cell wire model with dimensions in cm, including drain ports with vane cross-section, and **d** cropped image of test cell during single drain run with white dashed line outlining the vane.

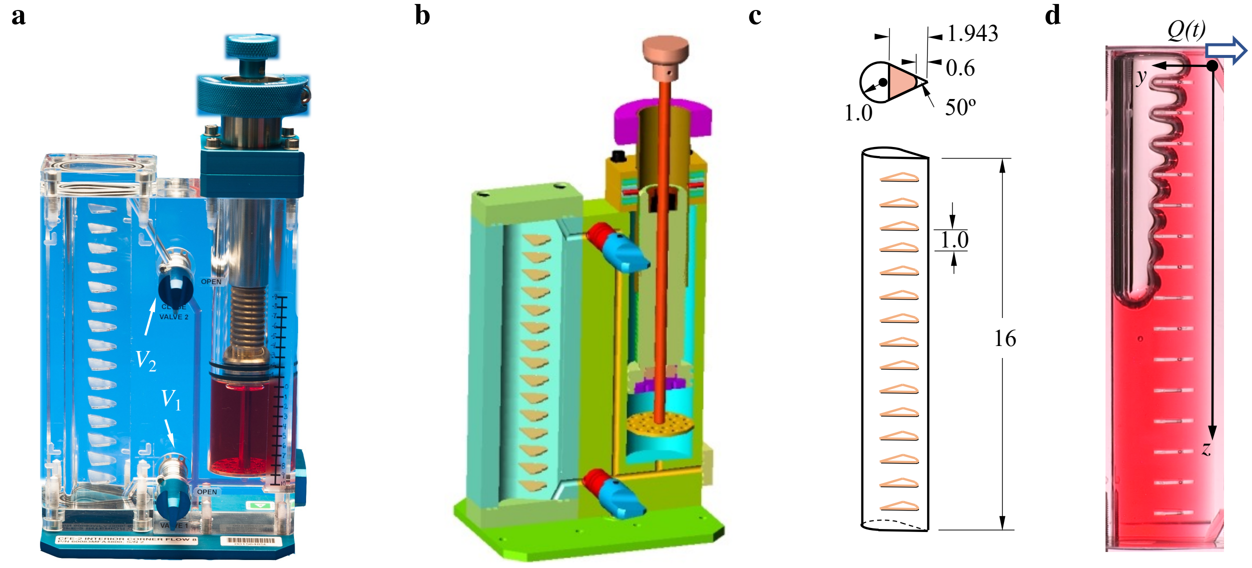

Supplementary Figure 7: ICF-8 test vessel: Partitioned ice-cream-cone section. **a** Test vessel, **b** solid model, **c** enlarged test cell wire model with dimensions in cm, and **d** cropped image of test cell single drain operation.

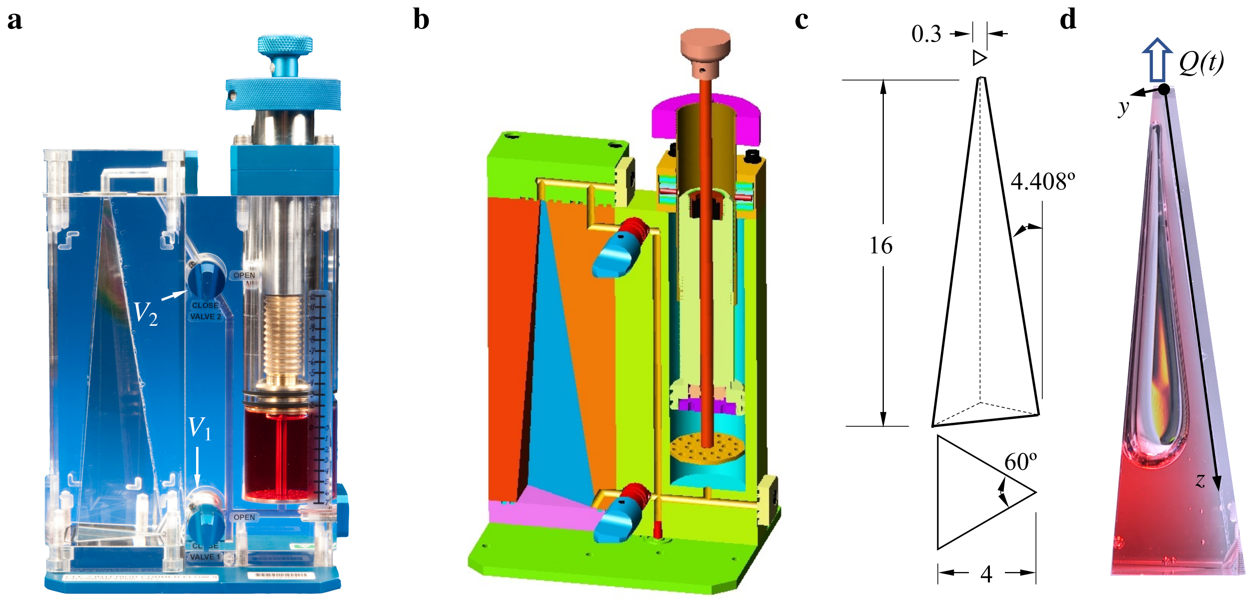

Supplementary Figure 8: ICF-9 test vessel: equilateral triangular pyramid. **a** Test vessel, **b** solid model, **c** enlarged test cell wire model with dimensions in cm, and **d** cropped image of test cell single drain operation.

Supplementary Table 1: ICF-2 fluid properties, scales, and constraints. Total of four identical corners $n = 4$.

**Property**	**Units**	**ICF-2 Small**	**ICF-2 Big**
Density, ρ	kg m^{-3}	839	839
Viscosity, μ	kg m^{-1} s^{-1}	0.0017	0.0017
Surface tension, σ	N m^{-1}	0.0180	0.0180
Contact angle, θ	deg	0°	0°
**Scales**	**Units**	**ICF-2 Small**	**ICF-2 Big**
Half angle, α	deg	45°	45°
Flow length, L	mm	73, 76, 88	30
Height, H	mm	1.51, 1.53, 1.59	1.63
Perimeter, P_s	mm	66, 67, 75	81
Surface area, A_s	mm^2	230, 240, 276	305
Geometry, F_i	-	1/7	1/7
Velocity, $W = \sigma \sin^2 \alpha F_i/\mu f$	mm s^{-1}	313	313
Flow rate, $Q = 4Q_j = 4WF_A H^3/L$	mm^3 s^{-1}	18.7, 18.6, 17.9	14
Time, $t \sim A_s L/Q$	s	225, 225, 245	41
Time offset, t_0	-	3/2	3/2
**Lubrication Assumptions**	**Constraint**	**ICF-2 Small**	**ICF-2 Big**
Slender geometry, $\epsilon = H/L$	$\epsilon^2 \ll 1$	$\sim 10^{-4}$	0.0029
Capillary dominance	$Bo \ll 1$	$\sim 10^{-4}$	$\sim 10^{-4}$
Low streamwise curvature	$\epsilon^2 f \ll 1$	0.0010	0.0071
Low intertia	$\epsilon^2 \rho\sigma H \sin^4 \alpha/(f\mu^2) \ll 1$	0.354	2.60
Low normal stress	$\epsilon^2 \sin^2 \alpha \ll 1$	$\sim 10^{-4}$	0.0015
Low saturation limit	$\beta/(1-\beta) \ll 1$	0.0127	0.0110
Static CL	$\epsilon\beta \sin^2 \alpha/f \ll 1$	$\sim 10^{-5}$	$\sim 10^{-4}$
Concus-Finn wetting	$\theta < 90° - \alpha$	satisfied	satisfied

Supplementary Table 2: ICF-3 fluid properties, scales, and constraints for $n = 1$ corner.

**Property**	**Units**	**ICF-3 Small**	**ICF-3 Big**
Density, ρ	kg m^{-3}	839	839
Viscosity, μ	kg m^{-1} s^{-1}	0.0017	0.0017
Surface tension, σ	N m^{-1}	0.0180	0.0180
Contact angle, θ	deg	$0°$	$0°$
**Scales**	**Units**	**ICF-3 Small**	**ICF-3 Big**
Half angle, α	deg	$45°$	$45°$
Flow length, L	mm	40	53
Height, H	mm	2.63	1.89
Perimeter, P_s	mm	107	64.3
Surface area, A_s	mm^2	672	289
Geometry, F_i	-	1/7	1/7
Velocity, $W = \sigma \sin^2 \alpha F_i / \mu f$	mm s^{-1}	313	313
Flow rate, $Q \sim W F_A H^3 / L$	mm^3 s^{-1}	178	50.1
Time, $t \sim A_s L / Q$	s	151	306
Time offset, t_0	-	3/2	3/2
**Lubrication Assumptions**	**Constraint**	**ICF-3 Small**	**ICF-3 Big**
Slender geometry, $\epsilon = H/L$	$\epsilon^2 \ll 1$	0.0043	0.0013
Capillary dominance	$Bo \ll 1$	$\sim 10^{-4}$	$\sim 10^{-4}$
Low streamwise curvature	$\epsilon^2 f \ll 1$	0.0104	0.0031
Low intertia	$\epsilon^2 \rho \sigma H \sin^4 \alpha / (f \mu^2) \ll 1$	6.16	1.31
Low normal stress	$\epsilon^2 \sin^2 \alpha \ll 1$	0.0022	$\sim 10^{-4}$
Low saturation limit	$\beta/(1-\beta) \ll 1$	0.0131	0.0157
Static CL	$\epsilon \beta \sin^2 \alpha / f \ll 1$	$\sim 10^{-4}$	$\sim 10^{-4}$
Concus-Finn wetting	$\theta < 90° - \alpha$	satisfied	satisfied

Supplementary Table 6: ICF-9 fluid properties, scales, and constraints for $n = 3$ identical corners.

**Property**	**Units**	**ICF-9**
Density, ρ	kg m^{-3}	950
Viscosity, μ	kg m^{-1} s^{-1}	0.019
Surface tension, σ	N m^{-1}	0.0206
Contact angle, θ	deg	$0°$
**Scales**	**Units**	**ICF-9**
Half angle, α	deg	$30°$
Flow length, L	mm	94
Height, H	mm	4.5
Perimeter, P_s	mm	84
Surface area, A_s	mm^2	342
Geometry, F_i	-	0.129
Velocity, $W = \sigma \sin^2 \alpha F_i / \mu f$	mm s^{-1}	35
Flow rate, $Q = 3Q_j = 3 W F_A H^3 / L$	mm^3 s^{-1}	73
Time, $t \sim A_s L / Q$	s	438
Time offset, t_0	-	3
**Lubrication Assumptions**	**Constraint**	**ICF-9**
Slender geometry, $\epsilon = H/L$	$\epsilon^2 \ll 1$	0.0024
Capillary dominance	$Bo \ll 1$	$\sim 10^{-4}$
Low streamwise curvature	$\epsilon^2 f \ll 1$	0.0024
Low intertia	$\epsilon^2 \rho \sigma H \sin^4 \alpha / (f \mu^2) \ll 1$	0.0369
Low normal stress	$\epsilon^2 \sin^2 \alpha \ll 1$	$\sim 10^{-4}$
Low saturation limit	$\beta/(1-\beta) \ll 1$	0.0435
Static CL	$\epsilon \beta \sin^2 \alpha / f \ll 1$	$\sim 10^{-4}$
Concus-Finn wetting	$\theta < 90° - \alpha$	satisfied

Supplementary Table 3: ICF-4 fluid properties, scales, and constraints for $n = 1$ corner.

**Property**	**Units**	**ICF-4 Small**	**ICF-4 Big**
Density, ρ	kg m^{-3}	839	839
Viscosity, μ	kg m^{-1} s^{-1}	0.0017	0.0017
Surface tension, σ	N m^{-1}	0.0180	0.0180
Contact angle, θ	deg	$0°$	$0°$
**Scales**	**Units**	**ICF-4 Small**	**ICF-4 Big**
Half angle, α	deg	$25°$	$25°$
Flow length, L	mm	70	70,60
Height, H	mm	6.80	7.30
Perimeter, P_s	mm	77.9	83.6
Surface area, A_s	mm^2	362	418
Geometry, F_i	-	0.133	0.133
Velocity, $W = \sigma \sin^2 \alpha F_i / \mu f$	mm s^{-1}	343	344
Flow rate, $Q \sim W F_A H^3 / L$	mm^3 s^{-1}	835	1034,1206
Time, $t \sim A_s L / Q$	s	30.4	28.3,20.8
Time offset, t_0	-	3/2	3/2
**Lubrication Assumptions**	**Constraint**	**ICF-4 Small**	**ICF-4 Big**
Slender geometry, $\epsilon = H/L$	$\epsilon^2 \ll 1$	0.0094	0.0109, 0.0148
Capillary dominance	$Bo \ll 1$	$\sim 10^{-4}$	$\sim 10^{-4}$
Low streamwise curvature	$\epsilon^2 f \ll 1$	0.0069	0.0080,0.0108
Low intertia	$\epsilon^2 \rho \sigma H \sin^4 \alpha / (f \mu^2) \ll 1$	14.6	18.1,24.6
Low normal stress	$\epsilon^2 \sin^2 \alpha \ll 1$	0.0017	0.0019,0.0026
Low saturation limit	$\beta / (1 - \beta) \ll 1$	0.0741	0.0741
Static CL	$\epsilon \beta \sin^2 \alpha / f \ll 1$	0.0016	0.0018,0.0020
Concus-Finn wetting	$\theta < 90° - \alpha$	satisfied	satisfied

Supplementary Table 4: ICF-6 fluid properties, scales, and constraints for both $\alpha_1 = 22.5°$ and three $\alpha_2 = 45°$ corners, a total of $n = 5$.

**Property**	**Units**	**ICF-6 Open**	**ICF-6 vane**
Density, ρ	kg m^{-3}	910	910
Viscosity, μ	kg m^{-1} s^{-1}	0.0045	0.0045
Surface tension, σ	N m^{-1}	0.0197	0.0197
Contact angle, θ	deg	0°	0°
**Scales**	**Units**	**ICF-6 Open**	**ICF-6 vane**
Half angle, α_1	deg	22.5°	22.5°
Flow length, L	mm	53	63, 55
Height, H_1	mm	8.8	8.8
Perimeter, P_s	mm	80	80
Surface area, A_s	mm^2	400	400
Geometry, $F_{i\,1}$	-	0.135	0.135
Velocity, $W_1 = \sigma \sin^2 \alpha_1 \; F_{i\,1} \, / \mu f_1$	mm s^{-1}	140	140
Flow rate, $Q = \sum_j Q_j \sim W_j \; F_{A\,j} \, H_j^3/L$	mm^3 s^{-1}	1765	1485, 1700
Time, $t \sim A_s L/Q$	s	12.0	17.0, 13.0
Time offset, t_0	-	3/2	3/2
**Lubrication Assumptions**	**Constraint**	**ICF-6 Open**	**ICF-6 vane**
Slender geometry, $\epsilon_1 = H_1/L$	$\epsilon_1^2 \ll 1$	0.0276	< 0.0257
Capillary dominance	$Bo \ll 1$	$\sim 10^{-4}$	$\sim 10^{-4}$
Low streamwise curvature	$\epsilon_1^2 f_1 \ll 1$	0.0171	< 0.0159
Low intertia	$\epsilon_1^2 \rho\sigma H_1 \sin^4 \alpha_1/(f_1 \mu^2) \ll 1$	7.5	5.3, 6.9
Low normal stress	$\epsilon_1^2 \sin^2 \alpha_1 \ll 1$	0.004	< 0.0038
Low saturation limit	$\beta_1/(1 - \beta_1) \ll 1$	0.1015	0.1015
Static CL	$\epsilon_1 \beta_1 \sin^2 \alpha_1/f_1 \ll 1$	0.0036	< 0.0035
Concus-Finn wetting	$\theta < 90° - \alpha_1$	satisfied	satisfied

Supplementary Table 5: ICF-8 fluid properties, scales, and constraints for $n = 1$ corner. All five runs identical.

**Property**	**Units**	**ICF-8**
Density, ρ	kg m^{-3}	910
Viscosity, μ	kg m^{-1} s^{-1}	0.0045
Surface tension, σ	N m^{-1}	0.0197
Contact angle, θ	deg	$0°$
**Scales**	**Units**	**ICF-8**
Half angle, α	deg	$25°$
Flow length, L	mm	60
Height, H	mm	7.3
Perimeter, P_s	mm	83.6
Surface area, A_s	mm^2	418
Geometry, F_i	-	0.133
Velocity, $W = \sigma \sin^2 \alpha F_i / \mu f$	mm s^{-1}	142
Flow rate, $Q \sim W F_A H^3 / L$	mm^3 s^{-1}	499
Time, $t \sim A_s L / Q$	s	50.2
Time offset, t_0	-	0
**Lubrication Assumptions**	**Constraint**	**ICF-8**
Slender geometry, $\epsilon = H/L$	$\epsilon^2 \ll 1$	0.0148
Capillary dominance	$Bo \ll 1$	$\sim 10^{-4}$
Low streamwise curvature	$\epsilon^2 f \ll 1$	0.0108
Low intertia	$\epsilon^2 \rho \sigma H \sin^4 \alpha / (f \mu^2) \ll 1$	4.17
Low normal stress	$\epsilon^2 \sin^2 \alpha \ll 1$	0.0026
Low saturation limit	$\beta / (1 - \beta) \ll 1$	0.0741
Static CL	$\epsilon \beta \sin^2 \alpha / f \ll 1$	0.0020
Concus-Finn wetting	$\theta < 90° - \alpha$	satisfied

There are 27 ISS container drains that are each uploaded as MATLAB .m data files. Each file contains dimensional results, corrected for optical distortion, but not smoothed. Each file name indicates the ICF test cell that data file corresponds to. There are 5 data components within each .m file:

1. `t`: time (s), $N \times 1$ vector
2. `piston`: piston location with arbitrary initial position (mm), $N \times 1$ vector
3. `h`: meniscus height from primary interior corner (mm), $M \times N$ vector
4. `z`: meniscus downstream location from drain (mm), $M \times 1$ vector
5. `z2`: bubble front location downstream from drain (mm), $N \times 1$ vector.

Supplementary Figure 1 is a snapshot of the ICF-4 vessel, and overlays `h` (black) for the entire binned region domain `z`. Then `h` is not valid along the entire `z` domain, but only up to the advancing front z_2. For this reason, `z2` location is provided (green) to aid in data analysis. Shown also is the piston location `piston` (blue). Volumetric flow rates are obtained by taking time-differences of `piston` and multiplying by piston area $\pi 19^2$ (mm^2).

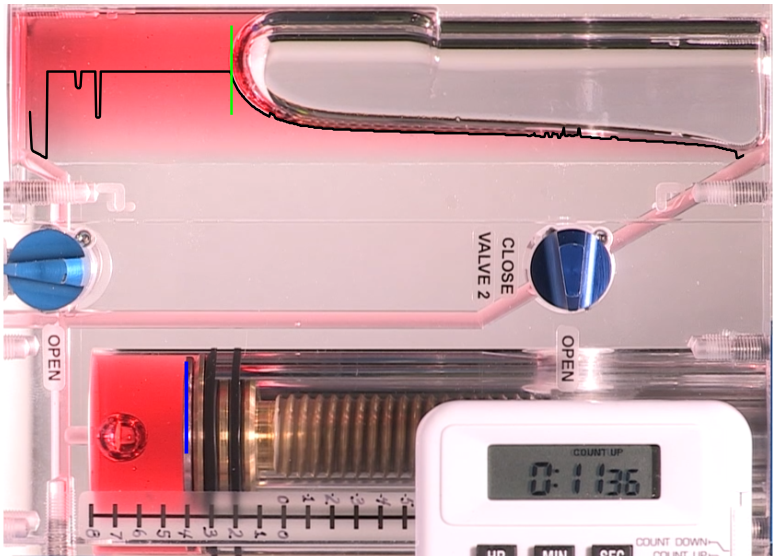

Supplementary Figure 10: ICF-4 test vessel, plotting interfacial height `h` (black), bulk location z_2 (green line) and piston location `piston` (blue line).
